# Ten steps or climbing a mountain: A study of Australian health professionals' perceptions of implementing the baby friendly health initiative to protect, promote and support breastfeeding

**DOI:** 10.1186/1472-6963-11-208

**Published:** 2011-08-31

**Authors:** Virginia Schmied, Karleen Gribble, Athena Sheehan, Christine Taylor, Fiona C Dykes

**Affiliations:** 1 School of Nursing and Midwifery, University of Western Sydney, Sydney, Australia; 2 Faculty of Nursing and Health, Avondale College, NSW, Australia; 3 School of Health, University of Central Lancashire, England

**Keywords:** Baby Friendly Health Initiative, breastfeeding, midwifery, health services research, dissemination of innovation, translational research

## Abstract

**Background:**

The Baby Friendly Hospital (Health) Initiative (BFHI) is a global initiative aimed at protecting, promoting and supporting breastfeeding and is based on the ten steps to successful breastfeeding. Worldwide, over 20,000 health facilities have attained BFHI accreditation but only 77 Australian hospitals (approximately 23%) have received accreditation. Few studies have investigated the factors that facilitate or hinder implementation of BFHI but it is acknowledged this is a major undertaking requiring strategic planning and change management throughout an institution. This paper examines the perceptions of BFHI held by midwives and nurses working in one Area Health Service in NSW, Australia.

**Methods:**

The study used an interpretive, qualitative approach. A total of 132 health professionals, working across four maternity units, two neonatal intensive care units and related community services, participated in 10 focus groups. Data were analysed using thematic analysis.

**Results:**

Three main themes were identified: 'Belief and Commitment'; 'Interpreting BFHI' and 'Climbing a Mountain'. Participants considered the BFHI implementation a high priority; an essential set of practices that would have positive benefits for babies and mothers both locally and globally as well as for health professionals. It was considered achievable but would take commitment and hard work to overcome the numerous challenges including a number of organisational constraints. There were, however, differing interpretations of what was required to attain BFHI accreditation with the potential that misinterpretation could hinder implementation. A model described by Greenhalgh and colleagues on adoption of innovation is drawn on to interpret the findings.

**Conclusion:**

Despite strong support for BFHI, the principles of this global strategy are interpreted differently by health professionals and further education and accurate information is required. It may be that the current processes used to disseminate and implement BFHI need to be reviewed. The findings suggest that there is a contradiction between the broad philosophical stance and best practice approach of this global strategy and the tendency for health professionals to focus on the ten steps as a set of tasks or a checklist to be accomplished. The perceived procedural approach to implementation may be contributing to lower rates of breastfeeding continuation.

## Background

Breastfeeding is essential to the normal health, growth and development of infants and early termination of breastfeeding is associated with adverse health consequences for both infants and their mothers [1,2]. The World Health Organization (WHO) and the United Nations Children's Fund (UNICEF) have implemented a number of strategies to protect and promote breastfeeding globally. One of these strategies, launched in 1991-1992 is the Baby Friendly Hospital Initiative (BFHI) now known in Australia as the Baby Friendly Health Initiative. The BFHI is aimed at promoting and supporting breastfeeding and is based on ten best practice standards [3] that must be attained in order for a hospital or health service to be accredited as 'Baby Friendly' (see table 1) [3]. Administration of the BFHI is carried out at the national level and, worldwide, there are over 20,000 BFHI accredited facilities. There is increasing evidence that implementation of the BFHI increases initiation and, to a lesser extent, duration rates of any breastfeeding and exclusive breastfeeding although individual studies show variable effects at different time points [4-7].

**Table 1:** Ten Steps to Successful Breastfeeding [3]

Every facility providing maternity services and care for newborn infants should:
1. Have a written breastfeeding policy that is routinely communicated to all health care staff.
2. Train all health care staff in skills necessary to implement this policy.
3. Inform all pregnant women about the benefits and management of breastfeeding.
4. Help mothers initiate breastfeeding within half an hour of birth.
5. Show mothers how to breastfeed, and how to maintain lactation even if they should be separated from their infants.
6. Give newborn infants no food or drink other than breast milk, unless medically indicated.
7. Practise rooming-in-that is, allow mothers and infants to remain together-24 hours a day.
8. Encourage breastfeeding on demand.
9. Give no artificial teats or pacifiers (also called dummies or soothers) to breastfeeding infants.
10. Foster the establishment of breastfeeding support groups and refer mothers to them on discharge from the hospital or clinic.

Currently, in Australia, breastfeeding initiation rates are high with around 90% of women initiating breastfeeding, however, exclusive breastfeeding rates drop rapidly following discharge from hospital [8,9]. The Australian National Health and Medical Research Council (NHMRC) list breastfeeding as a priority recommendation in dietary guidelines supporting exclusive breastfeeding for the first six months of life. At state level, New South Wales (NSW) Health has developed a breastfeeding policy that recommends the implementation of BFHI in all Area Health Services (AHS). To date, however, only 77 hospitals in Australia have been accredited as Baby Friendly [10] and in NSW there are only nine accredited hospitals [10], that is approximately 20% of hospitals with maternity units.

Few studies have investigated the facilitators or barriers to the implementation of BFHI. Factors such as strong support from the government and medical profession [11], credible leadership [12] and the presence of an organised central coordinating group [11,13] are reported as facilitators, while barriers include the investment of resources required for change management [14], inconsistency in the way policies are communicated and difficulties in educating staff [15] and the onerous nature of accreditation [13].

There is little research exploring the perceptions of health professionals towards the implementation of BFHI. Research in the facilitation of change suggests there are difficulties in implementing broad policy changes [16] and resistance to policy and practice change [17]. Greeenhalgh et al. [18], in a systematic review of the diffusion of innovations in service organisations, found that innovations perceived to be advantageous, compatible, and simple to use influenced the interaction between the innovation and its adoption by members of the organisation. This paper describes the perceptions that midwives and nurses have about the BFHI and examines factors that may facilitate or hinder the implementation process.

## Methods

This was an interpretive study utilising a qualitative method (focus groups) to elicit the perceptions of midwives, nurses, and clinical leaders with regard to the BFHI. Ethics approval was obtained from the relevant Human Research Ethics Committees prior to the commencement of the study. All participants were provided with written information about the research describing the purpose of the study and what participation would involve. All participants gave written consent. Participant confidentiality and autonomy were protected at all stages.

### Study Setting

The AHS in NSW, where the review took place, has a population of approximately one million residents and one of the highest birth rates in NSW (over 16,000 births per year). Notable socio-economic differences exist including certain localities with high levels of socio-economic disadvantage. The AHS has six publicly funded maternity units and two neonatal intensive care units.

### Study Participants and Recruitment

Study participants were recruited using both purposive and convenience sampling. A total of 132 health professionals, working across four maternity units, two neonatal intensive care units and related community services, participated in the study. Information about the study and the date and time for the focus groups were distributed to staff two weeks prior to the groups taking place. Participants were drawn from those who were willing and available to participate on the day of the group. Participants included 38 midwives and five student midwives working across four hospital-based maternity units, 20 neonatal nurses working in two neonatal units, and 45 child and family health nurses working in the community in one AHS in NSW, Australia. In addition, a purposive sample of 20 clinical leaders who were members of the AHS infant feeding coordination group participated in the study. The clinical leaders were predominantly midwifery and child and family health nursing managers and clinical consultants and were identified as participants because of their role in preparing policies related to infant feeding practices and facilitating implementation of the BFHI. Of the midwives and nurses who participated in the study, 20 percent had five years or less experience in practice as a midwife, neonatal nurse or child and family health nurse, 35 percent had between 5 and 15 years experience, and 45 percent had over 15 years experience, Thirty percent had qualifications as lactation consultants, and a further 30% had completed additional breastfeeding education facilitated within the AHS. All members of the infant feeding coordination group had International Board Certified Lactation Consultant qualifications.

### Data Collection

Data were collected using focus groups with midwives, neonatal nurses, and child and family health nurses. A total of 10 focus groups were held. Five focus groups comprised of midwives working in the maternity units that participated in the study, two focus groups with neonatal nurses working in the two participating nurseries, two groups with community based child and family health nurses and one focus group with the AHS infant feeding coordination group. The infant feeding group also has representation from nutritionists, health promotion officers, paediatricians, and a consumer representative from the Australian Breastfeeding Association. These members, however, were not present on the day and therefore did not participate in the focus group discussion.

All authors except FD participated in data collection with two team members present at each focus group. Each focus group lasted approximately one hour in duration and, with one exception, comprised between 6 to 12 participants. The exception was a community based child and family health nurses' group that was conducted during a scheduled in-service time and had 30 participants. This group was facilitated as a large group discussion. Questions used in the focus groups are outlined in Table 2. All focus groups were digitally recorded and transcribed verbatim.

**Table 2:** Focus groups/interviews-Key prompts

1. What do you know about the BFHI? What are your general views and opinions about the BFI?
2. What stage of BFI implementation is your unit at and what is your role in relation to the implementation of the BFI?
3. What do you think are the challenges that your hospital is or will face in becoming Baby Friendly? What aspects of the BFI do you think are, or will be easy for your hospital to implement? What aspects of the BFI do you think are or will be difficult?
4. If there was something you could change in how women are cared for with regards breastfeeding what would you change?
5. How do you think working in this hospital once it is baby friendly hospital will be different from what it's like working here now?
6. How do you think the experience of having a baby in this hospital will be different once becomes baby friendly?
7. Do you have a breastfeeding policy here? If so, how was it developed and do you refer to the breastfeeding policy? Do other staff, in general follow it? Why, why not?

### Data Analysis

Data were analysed using thematic analysis. This was an iterative and inductive process which involved listening to the recorded data, multiple readings of the transcribed data, identification and labeling codes in the data, and development of preliminary themes and subthemes. Themes and subthemes are represented in this paper using phrases and where appropriate using the language of the participants. Further coding of the data in each theme was undertaken, identifying linkages between themes [19].

## Results

Overall participants in this study perceived the BFHI in a positive way and were committed to BFHI implementation. Two of the participating maternity units reported that much progress had been made towards implementing the BFHI particularly within the respective maternity units. One group reported '*we are almost there' (FG 4) *and another group stated '*we have been doing Baby Friendly for years*' (FG3). Thematic analysis of data has resulted in three key themes: 'Belief and Commitment'; 'Interpreting BFHI' and 'Climbing a Mountain'.

### Belief and Commitment

Participants in this study were committed to the principles of BFHI, believed it was achievable and were mostly keen to gain BFHI accreditation. The BFHI was described as an evidence-based strategy that would lead to increased initiation and duration of breastfeeding: one participant stated, '*evidence has shown us that this is the right way to go' (FG1)*. Others spoke for their colleagues indicating commitment to the initiative, for example '*I think midwives here are 100 percent behind it' (FG4)*. BFHI was viewed as a strategy whose time had come and participants reflected on the achievements of other hospitals across Australia that had received accreditation and one participant commented '*Just looking at the ten steps... it is achievable*' (FG6).

#### Healthier babies

The study participants believed there were many benefits to receiving BFHI accreditation. In particular, they spoke of the health benefits to infants and women that would ensue from increased breastfeeding rates as well as the benefits to society from healthier children and later as adults:

*If you breastfeed your baby, it doesn't just have benefits here and now, it has benefits for the whole community further along the track. Then that also impacts on how the country develops as a nation and then it snowballs into looking at how everything works in the world (FG 1)*.

#### Pleasing women and the community

Participants in most groups were certain that, if implemented, BFHI would result in better antenatal preparation and consistency in approach and information provided by health professionals. Participants argued that these factors had the potential to improve the view that women and the community held about maternity services:

*It would be nice to be known out in the community as, oh they're really great, I got so much support there, they were really good, they're very positive for breastfeeding and I felt really comfortable, and I wouldn't have been able to breastfeed unless it was for all the staff at the hospital. instead of all the negative things that you hear-I got 150 different opinions on how to do this and that *(FG 1).

#### Happier staff

Importantly for the participants, one of the effects of this consistency in information was that it would also benefit staff:

*It will be less stressful...Because of always having to repeat the same thing over and over, and correct other people's wrong advice without actually telling them that midwife has given them wrong advice per se (FG 1.)*.

### Interpreting BFHI

In each focus group participants were asked to describe the BFHI and they were asked how they would explain this strategy to a novice. The responses were diverse. For some the BFHI was explained as a strategy that was to assist in delivering a key message about the benefits of breastfeeding and others focused on the potential for global health benefits of the BFHI. Most commonly however, explanations of the BFHI related to the Ten Steps to Successful Breastfeeding. There was also concern among participants that some midwives, nurses and other professionals misinterpreted the BFHI.

#### There is one key message

In two focus groups participants emphasised the importance of BFHI in delivering one key message: '*breastfeeding is the normal way to feed a baby' *(FG 10).

*But it's going to take a couple of generations anyway because the whole idea of Baby Friendly is to change the whole focus that breastfeeding is not a choice, that its natural and formula feeding is just a legitimate choice if that can't be carried out (FG 1)*.

This powerful message implies that BFHI is not just about changing health professional practice but also about changing women's views and practices and this would take a long time

#### A global view

In one focus group participants attempted to place the BFHI within the broader context as a global strategy to protect promote and support breastfeeding *'...the big BFH initiative is good in that it promotes and supports breastfeeding'*, (FG 1) and another participant in this group added:

*I think that's what the role of the World Health Organization is to do, it's there to promote health within the communities and the countries all over the earth and then it's to filter down to each specific community (FG 1)*.

#### There are ten steps to Baby Friendly!

In describing BFHI the majority of focus groups participants listed the ten steps to successful breastfeeding and expressed the belief that all components or steps had to be implemented:

*So it starts with antenatal education and finishes with referring them out to the community and work through all the steps of early breastfeeding (FG 5)*.

*Breastfeeding as much as possible without the use of dummies, bottles. Promotion of skin to skin contact directly after birth (FG1)*.

The BFHI was also interpreted in a simplistic way. For example, when discussing how one would recognise a 'Baby Friendly' hospital, one participant described it:

*We're very Baby Friendly because we've got pictures of boobs on our wall. As you walk down there, people breast feeding saying protecting from respiratory disease (FG 3)*.

#### Misinterpretation of BFHI

Participants in some groups, most particularly the infant feeding coordination group, were concerned that midwives, nurses, and other health professionals sometimes misinterpreted the intentions or meaning of the BFHI. For example, the following discussion in one focus group indicated that some of the senior staff, who were members of the infant feeding coordination group, were concerned that there were midwives and other staff who continued to believe that the BFHI is something that is being forced on staff and women:

*FG Participant 1: I still feel that there's a view out there that it's (BFHI) a fanatical way of operating*,

*FG Participant 2: I don't know whether the title maybe does it, to be honest*.

*FG Participant 1: Because to be Baby Friendly is just making sure that the mother has choices, and informed choices. There's nothing forced about it. It's actually stopping the staff doing things against the policy, such as dummies and*.

*FG Participant 3: There's limits and restrictions on staff, but not on mothers*.

*FG Participant 4: Yeah, it's not actually on the mothers, but it's perceived by the staff, and then projected in that way (FG 10)*.

There was concern by some midwives that by adhering rigidly to the ten steps women may feel pressured to breastfeed:

*I have actually come to the point that we are imposing something on them because some of them really don't want to.... Yes, because I have actually seen some staff trying hard and no matter what that woman has to breastfeed. That's why I feel you're imposing something*. (FG 4).

In another instance, a participating midwife indicated that she used the global nature of the BFHI as an explanation as to why she could not give the parents formula for their infant:

*Actually a husband yesterday said to me, we were talking about inconsistent information and he said my wife wanted the baby to have extra fluid, she thought she'd made her sick and I said no, no. And he said but why can they get away with this? I said well you can't blame the postnatal ward I said it's a World Health Organization ruling that breastfeeding is encouraged *(FG 4).

There was also a perception that implementation of the BFHI was something that had to be done, a directive rather than a recommendation from the NSW Department of Health. The perception that implementation of BFHI was mandatory was seen positive by some participants as it ensured there would be management support for staff who were trying to implement it:

*So we wrote the policy to be a mandatory directive so that those people at the ground level had the top-down support. To be able to say we have been told we have to do this, so you (hospital management) need to support us (FG 8)*.

#### Some midwives break the rules

Participants also provided examples where they or other health professionals engaged in practices that would not meet BFHI standards. Some practices such as taking a baby into the nursery or crèche for two or three hours at night were rationalised as supporting a '*desperately tired' *mother. Some participants described some of the ten steps as *'rules' *that were too rigid and not supportive of individual women's needs. For example, one participant spoke of taking a baby into the nursery area for two hours during the night:

*The mother's absolutely exhausted and they do ask if you can take the baby for a couple of hours. Well technically you're not supposed to but if you do take the baby for a couple of hours at least she gets two hours sleep (FG 4)*.

Another spoke of seeing value in supporting a woman who wishes to use one bottle of formula stating that it, *'may be enough to keep them going' (FG 3)*.

### Climbing a mountain

No matter how committed and how much progress had already occurred, participating midwives and nurses in all focus groups were under no illusion that implementation and accreditation as a BFHI hospital or community would be easy. They emphasised that a shift in attitude or change in practice by staff would be required to implement BFHI. Participants also believed that women and the community would need to change their views about breastfeeding.

#### It is hard work

Participants had experienced resistance to the changes required to implement the BFHI but believed this was inevitable, '*there is always resistance to change*' (FG 10) and '*I think, like every human being, we fight change because we get in our comfort zone' (FG1*. Continual resistance however, was tiring and made the implementation of BFHI seem like hard work: *'sometimes it seems a very big mountain'; it's going to take a while to change' (FG2)*.

#### There is no time

Participating midwives and nurses were clear that one of the most important resources that they needed to implement the BFHI and to support women with breastfeeding was time:

*We do not have the time to sit with all these women for twenty minutes or half-an-hour. You just don't have the time. You're not a one-on-one and what happens is that people forget that you're not looking, if you've got four or five women that you're looking after, then it's not four people you're looking after, you're looking after eight or ten bodies. You can't do it. It means you look after one person or four or five other people are neglected (FG 1)*.

#### The quick fix

The pressure of time may lead some health professionals to take short cuts or seek a 'quick fix':

*Yes, but they need to sit with the mother, I have had a few people say that often the baby is being given formula, because it keeps them quiet. And if they have got ten women to look after a night, and the mother needs them to sit with her for an hour, then it is easier to give the baby some formula than to sit down and spend the time giving her the assistance that she needs (FG 5)*.

#### Institutional priorities

Participants believed that it was institutional priorities such as ensuring a steady 'patient flow' or 'freeing up bed-block" that increased the pressure on their time and often this meant they were not able to give women the time they needed:

*As long as the baby's taking a bottle somehow and they're getting the fluid into them, they're okay to be discharged and the breastfeeding is continued at home but I sort of actually wonder how many mothers (go home) expressing EBM (expressed breast milk) through a bottle (FG 1)*.

Participants argued that if time was spent now to assist women with breastfeeding that it would save the time of other health professionals in the long run due to the health benefits:

*They [staff] don't see how much [giving a baby infant formula] impacts them, they only see what they're doing at the time. And everyone is stretched for time and so it seems like hard work at the time but if they could see the bigger picture they'd realise that we're actually causing more work for ourselves because the babies have to come back as inpatients because they're unwell, because they're not protected against disease, or they come back and they're obese. It just spirals out of control- just for that short term getting it quickly sorted *(FG 1).

#### Some steps are easier than others

While participating midwives and nurses perceived that restrictions on their time meant it was difficult to provide women with the support and education needed, they reported that some steps such as skin to skin at birth (step 4) were easier to put in place:

*It's [skin to skin] a time saver in the delivery suite as well because if you have your mother and baby skin to skin, that baby is safe with the mother,..., and more likely to latch on itself. You can just leave your mother and baby there quite happily. So it's not a time consuming thing for us because we can just leave them together quite safely and happily *(FG 1).

Similarly 24 hour rooming-in (step 7) was a practice participants reported as already well established in this AHS and they indicated that it had been in place for a long time. They also indicated that it was relatively easy to advise women that dummies (pacifiers) were not supplied and their use was discouraged (step 9) and that parents needed to bring in their own formula if they wished to use it without there being a medical indication present (step 6).

## Discussion

This paper describes the perceptions of the BFHI held by a group of Australian midwives and nurses working in one AHS in NSW, Australia. The findings indicated that, in principle, participants considered the BFHI to be a high priority, an essential set of practices or innovation that would have positive benefits for babies and mothers both locally and globally as well as for health professionals. The perceptions that health professionals and others hold of health innovations such as the BFHI can influence implementation [18,20]. However, despite the overall positive perceptions of BFHI, no hospitals in this AHS had applied for BFHI accreditation and further, across Australia only around 23 percent of hospitals with maternity services have achieved this goal [13] although it is 20 years since BFHI was launched.

Researchers suggest there are barriers to any policy and practice change [18,20,21] including the implementation of strategies to promote and support breastfeeding [15,16,22,23]. Drawing on the work of Rogers [24], Greenhalgh [18] developed a model that suggests that the characteristics of an innovation are important in influencing adoption of the innovation or policy and practice change. They identified a number of characteristics influencing adoption (see Table 3) and many of these characteristics are evident in the findings of this study. In the following discussion the results will be explored utilising the work of Greenhalgh and colleagues [18] to examine the characteristics of the BFHI that may influence its adoption or implementation.

**Table 3:** Characteristic of the innovation influencing adoption (adapted from Greenhalgh et al[18])

**• relative advantage **- innovations that have a clear unambiguous advantage in either effectiveness or cost effectiveness are more easily adopted and implemented (p 594).
**• compatibility **- innovations that are compatible with the adopters norms, values, needs are more easily adopted; similarly if compatible with organisation's or professions' norms, values, ways of doing things the innovation will be more easily adopted (p. 596).
**• complexity **- innovations that are perceived by key players as simple to use are easier to adopt. Complexity can be reduced by practical experience and by demonstration or by breaking the innovation into manageable parts and adopted incrementally/If there are few organisational response barriers then it is easier to adopt an innovation (p.596).
**• trialability **- if the innovation can be trialled it will be more easily adopted (p. 596).
**• observability **- if benefits of innovation can be seen by adopters then it will be more easily adopted (p. 596).
**• reinvention **- if potential adapters can modify or refine the innovation to suit adopters and organisations then it will be more easily adopted (p.596).
**• fuzzy boundary **- innovations will typically have a hard core or elements that are non-negotiable) and a 'soft periphery' of organisational structures and systems that need to adapt to accommodate the innovation-the more adaptable the periphery, the easier it will be to adopt the innovation (.597).
**• risk **- if the innovation is surrounded by a high degree of uncertainty related to outcome then it is less likely to be adopted easily (p. 597).
**• task issues **- if an innovation is relevant to the user's work and if it makes a job easier then it is more easily adopted (p. 597).
**• knowledge required **- if knowledge required to use or impellent the innovation can be codified and transferred in different contexts, then it will be more easily adopted (p.597).
**• augmentation/support **- external support eg customization, training, will help increase adoption (p. 598).

### Study limitations

It is important to note the limitations of this study. This study was conducted in one AHS in NSW, Australia and participation was voluntary and limited to those midwives and nurses interested in attending and available at the scheduled date and time. Participants included two midwives who had worked previously in a BFHI accredited hospital and it may be that their experiences of BFHI could have influenced the views of those participants involved in implementation for the first time, either prior to or during the focus group. It may also be that the perceptions of staff working in hospitals that have BFHI accreditation differ from those who participated in this study. We also recognise that the perspectives of other health professionals who may influence BFHI implementation such as paediatricians, obstetricians, general practitioners, health promotion officers and peer support organisations have not been included in this study. The findings should therefore be interpreted with caution. The scope of this study has precluded interviewing women who are accessing these maternity services, but this is clearly an important perspective and will be the focus of ongoing research.

### The Innovation-Relative advantage and compatibility

The BFHI was seen as a way to promote and support breastfeeding thereby, improving the health of babies, pleasing women, families and the community, and making staff happier, suggesting the innovation was perceived as having a 'relative advantage' over current practice or other single interventions or strategies that may be effective in promoting and supporting breastfeeding [25,26]. Some participants reported they were influenced by the international research supporting the implementation of BFHI and they attributed increases in breastfeeding initiation and duration rates both locally and globally to the implementation of the BFHI.

In addition, given the professional and public acknowledgement of the importance of breastfeeding to the health of mothers and babies [27-29], an innovation such as the BFHI that supports breastfeeding is generally compatible with social and professional norms in Australia. Consequently, there appeared to be no doubt about whether the BFHI should be implemented, rather it was a matter of how, and within what time frame it could be implemented. It was considered achievable but would take commitment and hard work to overcome the numerous challenges and to 'climb the mountain' towards attaining BFHI accreditation.

### Trialability and Observability

Greenhalgh et al [18] also indicate that an innovation is more likely to be adopted if there is an observable benefit to doing so and if the innovation or aspects of it can be trialled prior to full implementation. Participants were heartened in their endeavours to implement BFHI because they were aware that other hospitals had been accredited in Australia. Furthermore, staff from two of the participating maternity units reported that all of the ten steps were already implemented within the maternity unit and with a little more work with other professionals and sections of the hospital they would be able to achieve BFHI accreditation. Both maternity units report breastfeeding rates at discharge from hospital of over 90 percent [30].

However, the 'observability' of the benefits of BFHI is yet to be established in the Australian context. While acknowledging the positive effect of the BFHI in increasing breastfeeding in countries with low initiation and duration rates [31-33], there is little evidence to date to suggest the implementation of the BFHI in Australia will have a positive effect on breastfeeding rates particularly increased duration rates [34,35]. Participants also noted that some of the outcomes of BFHI, for example the long term health impact of the minimisation of artificial feeding in hospital, would not be observable to those implementing BFHI and therefore decreases the impetus to implement BFHI.

### Complexity and task focused

The less complex an innovation is, the more likely it will be adopted. BFHI is a complex innovation, and the perceived complexity of implementing and evaluating BFHI is reflected in the number of studies that have trialled one or two components of the BFHI, for example the introduction of professional education to support breastfeeding [23,36] and skin to skin contact in the first hour after birth [37,38]. However, this indication of complexity also illustrates how the developers of BFHI used a classical simplification process of breaking the innovation down into more feasible parts (steps). This is demonstrated in the analysis where the participants discussed their achievements in implementing some of the steps and identifying where further work was required. For example, steps 4, 6, 7 and 9 related to the practice of skin to skin contact and restricting use of infant formula or pacifiers appeared easier to implement than steps 2, 5, 10 which involve staff having time to provide breastfeeding education and support for women (lack of time to to meet the information and support needs of breastfeeding women is common [39]).

### Reinvention and fuzzy boundaries

Greenhalgh et al [18] suggest that innovations that are flexible or able to be adapted to the particular needs of the organisation so that it meets the clients' needs or the needs of staff are more likely to be adopted. In this study some participants expressed concern that the BFHI represented a set of rules that health professionals had to apply in the same way in all settings, with all women and babies. According to the participants there appeared to be little space for reinvention or flexibility in service provision. Furber and Thomson [40], in the UK, also report that midwives 'break the rules' in order to support mothers in what they perceived as beneficence. The apparent inflexibility of BFHI may therefore be a barrier to its implementation.

### Augmentation support

Greenhalgh et al [18] indicate that an innovation will often need additional organisational support which may range from endorsement, to provision of additional resources to support implementation. This study found that while BFHI had endorsement at State level, participants were concerned that there was no additional organisational or institutional support for implementing BFHI; this meant that institutional priorities, such as freeing up 'bed block,' were more important than ensuring that a woman felt confident with breastfeeding before discharge. In this site, no one person or group had a mandate to implement the BFHI, which Walsh et al [13] believe is crucial to implementation and comprises part of the accreditation process. It is also difficult to ascertain the financial costs associated with BFHI implementation and accreditation and, to date, there has not been a cost benefit analysis of BFHI. The NICE guidelines on postnatal care [41] suggest that the cost of preparing a BFHI implementation plan and the initial assessment is approximately 6,000 UK pounds. This does not include the cost of a BFHI coordinator or the significant cost of ensuring all staff is trained.

### Adoption by Individuals- The meaning

There were differences in the perceptions and interpretations of BFHI across participants. Greenhalgh et al [18] stated that the meaning of an innovation to individuals was important to its adoption. Whether an individual's understanding of the innovation fits with the understandings of managers, service users, and other stakeholders will affect individual adopters. Different perceptions could potentially lead to unfulfilled expectations and disillusionment with the innovation. In this study some participants appeared to raise the status of the BFHI to that of a 'saviour' of breastfeeding and thereby a strategy that would increase health and well being of children and adults both locally and globally. For most participants, however, the BFHI was perceived as a list of tasks to be achieved.

Participants also tended to equate the BFHI with breastfeeding promotion. Rather than the BFHI being a strategy to improve the practices of health professionals within the hospital setting, it was interpreted as a strategy to convey the key message to mothers that 'breastfeeding is the normal way to feed a baby'. In this way the BFHI was seen as influencing infant feeding decisions. Some participants interpreted this to mean that women were given little choice in their feeding method and that they may be, or were being, pressured to breastfeed. Staff concerns about pressuring women to breastfeed has been reported by others [40] and many studies have reported women feeling pressured to breastfeed by midwives [28,29,42]. This lack of understanding of the BFHI could clearly lead to difficulties in BFHI adoption as individuals may be opposed to BFHI implementation based on their (mis)perceptions of what being 'Baby Friendly' involves rather than what it actually does involve.

In summary, the findings of this study were explored within the context of two aspects of Greenhalgh [18] model for diffusion of innovations-the innovation and the adoption by individuals. From the findings BFHI as an innovation has:

• relative advantage to midwives, mothers and babies

• apparent compatibility with the midwifery philosophy of practice

• complexity, but is broken down into steps for easier adoption

• trialability-it has already been adopted by others

• observability of its benefits has yet to be established in Australia

• limited reinvention-the BFHI was perceived as being non-modifiable

• clear not fuzzy boundaries although at time these boundaries appeared to be too rigid

• limited augmentation and support for the BFHI by the organisation and managers

Adoption by individuals:

• the meaning of the BFHI to individuals influenced its implementation, for example the BFHI was viewed as a saviour or a burden.

## Conclusion

The findings of this study suggest strong support from health professionals for the implementation of BFHI in the participating AHS. However, it is evident that the principles of this global strategy are interpreted differently by health professionals and that further education and accurate information about the BFHI is required. It may be that the current processes in place to disseminate and implement BFHI need to be reviewed. The findings suggest that there is a contradiction between the broad philosophical stance and best practice approach of this global strategy and the tendency for health professionals to focus on the ten steps as a set of tasks or a 'checklist' to be accomplished. Taking a procedural or bureaucratic approach to the implementation of BFHI may, in fact, contribute to lower rates of breastfeeding continuation in the first 8 weeks after birth. This needs further research.

## List of Abbreviation

BFHI: Baby Friendly Health Initiative; WHO: World Health Organization,; UNICEF: United Nations Children's Fund; NHMRC: National Health and Medical research Council; AHS: Area Health Service.

## Competing interests

The authors declare that they have no competing interests.

## Authors' contributions

VS led the conception and design of study, participated in the acquisition, analysis and interpretation of data and preparation and revision of the manuscript. KG played a major role in acquisition, analysis and interpretation of data, preparation and revision of the manuscript. AS made a substantial contribution to conception and design, interpretation of data and preparation and revision of the manuscript. CT participated in the acquisition of data, analysis and interpretation of data and critical revision of the manuscript. FD made substantial contributions to conception and design, interpretation of data and critical revision of manuscript. All authors have read and approved the final manuscript.

## Pre-publication history

The pre-publication history for this paper can be accessed here:

http://www.biomedcentral.com/1472-6963/11/208/prepub
